# The Private Clinic System in Great Britain

**Published:** 1921-06-25

**Authors:** 


### Hospital Facilities for the Well-to-Do.

### THE PRIVATE CLINIC SYSTEM IN GREAT BRITAIN

Before the Fellows of the Royal Society of Medicine on Thursday, June 16, Sir Thomas Horder, who has recently returned from America, read a paper on "The Problem of the Private Clinic System in Great Britain." Sir J. Bland Sutton was in the chair. Sir Thomas pointed out that the facilities for group practice which are afforded by general hospital conditions have led to increased efficiency so far as the patient is concerned, and to an advance in medical knowledge. The development of this system in private work is considerably overdue. Something is needed to replace the present unsatisfactory conditions in connection with nursing homes. A clinic system should be so planned as to secure that the ordinary routine physical examination of the patient is supplemented by providing facilities for expert investigations and for a more thorough and beneficial treatment than can be undertaken by one individual practitioner. Such a system would not only save time both for the doctor and the patient, but it would save money for the patient without the sacrifice of any efficiency. Certain private groups or clinics now existing or under consideration are out of court, but there are two forms of private clinic which operate to supply the desiderata. First, a number of practitioners chosen carefully to be representative of various branches of medicine; this is a true group. Secondly, a practitioner or consultant of great experience and balanced judgment who, after a careful routine examination and with the history, refers the patient to a specialist for expert examination and report. This fulfils the desiderata, but is not a true group. Both these systems are practicable, and should be encouraged. Sir Thomas then proceeded to discuss the relative advantages and disadvantages of the group clinic and the personal clinic and the difficulties inherent in each. He summed up that the private clinic supervised by one man has up till now been more easy to set going in this country than the group clinic. Great Britain is not the United States, and British patients are not American. All the same, it seems highly probable that the group clinic will soon become a successful British institution. Group medicine is, however, better adapted to urban than to rural conditions. Sir Thomas was followed by Sir Humphry Rolleston, Dr. Drury Pennington, Mr. Bishop Harman, Mr. Clayton Greene, Mr. W. S. Dickie, Dr. G. C. Anderson (deputy medical secretary British Medical Association), and Dr. A. F. Hurst. Each dealt with various aspects of the question, but the expressed opinion of all the speakers was markedly in favour of the establishment in this country of private clinics, which shall provide for the well-to-do and the moderately endowed an alternative to and an improvement on the private nursing home, and shall indeed supply all the facilities in illness which at present can only be obtained through admission to the wards of a large general hospital. Mr. W. S. Dickie gave some interesting figures concerning the material or financial side of the establishment of a clinic system. He maintains that the cost of building such a clinic, to include the necessary equipment for making all types of examinations and investigations, need not exceed £500 per bed, and in this connection he protested against over-elaboration and luxury; simplicity should be the dominating note. The running expenses per bed in a private hospital would be £3 per week, and reckoning a return of 10 per cent. on the invested capital, he calculates that at a charge of £7 7s. a week such a clinic could be made to pay. Other speakers discussed the financial relations of the members of the clinic, and the general view, which was voiced by Dr. Anderson and others, was that such clinics should be entirely in the hands of the medical profession, that there should be no lay control, and that they should not be advertised. A private clinic should be conducted on lines to secure the co-operation of the general practitioner and the consulting branch of the profession.

The meeting, which lasted for two hours, was adjourned to a later date, so that full opportunity for discussion might be given to all the Fellows.

